# Therapeutic Targeting of DNA Replication Stress in Cancer

**DOI:** 10.3390/genes14071346

**Published:** 2023-06-26

**Authors:** Long Gu, Robert J. Hickey, Linda H. Malkas

**Affiliations:** 1 Department of Molecular Diagnostics & Experimental Therapeutics, Beckman Research Institute of City of Hope, Duarte, CA 91010, USA; lmalkas@coh.org; 2 Department of Cancer Biology & Molecular Medicine, Beckman Research Institute of City of Hope, Duarte, CA 91010, USA; rohickey@coh.org

**Keywords:** replication stress, chemotherapy, proliferating cell nuclear antigen, DNA repair, cancer

## Abstract

This article reviews the currently used therapeutic strategies to target DNA replication stress for cancer treatment in the clinic, highlighting their effectiveness and limitations due to toxicity and drug resistance. Cancer cells experience enhanced spontaneous DNA damage due to compromised DNA replication machinery, elevated levels of reactive oxygen species, loss of tumor suppressor genes, and/or constitutive activation of oncogenes. Consequently, these cells are addicted to DNA damage response signaling pathways and repair machinery to maintain genome stability and support survival and proliferation. Chemotherapeutic drugs exploit this genetic instability by inducing additional DNA damage to overwhelm the repair system in cancer cells. However, the clinical use of DNA-damaging agents is limited by their toxicity and drug resistance often arises. To address these issues, the article discusses a potential strategy to target the cancer-associated isoform of proliferating cell nuclear antigen (caPCNA), which plays a central role in the DNA replication and damage response network. Small molecule and peptide agents that specifically target caPCNA can selectively target cancer cells without significant toxicity to normal cells or experimental animals.

## 1. Introduction

A hallmark of malignancy is the enhanced rate of spontaneous DNA damage due to compromised DNA replication machinery in cancer cells [1,2]. In addition, the enhanced metabolic activity of cancer cells creates an elevated level of reactive oxygen species (ROS), which can cause DNA damage. The loss of tumor suppressor genes or constitutive activation of oncogenes can also elicit substantial DNA damage, further exacerbating DNA replication stress in cancer cells [3]. Whereas acquired genomic alteration is responsible for tumor initiation and progression, which leads to more aggressive sub clones [4], it also provides a key cancer vulnerability for therapeutic intervention. Lesions on DNA templates frequently obstruct replication machinery and, if not resolved, cause the collapse of DNA replication forks, leading to lethal double-stranded DNA breaks (DSBs) and apoptosis. To support survival and proliferation and to maintain genome stability, cancer cells are intrinsically addicted to DNA repair machinery and signaling pathways [5,6]. It is not surprising that many chemotherapeutic drugs exploit the genetic instability of cancer cells by overloading replication stress. This concept of targeting DNA for chemotherapy has led to the development of numerous anticancer compounds over almost six decades. Based on the mechanisms, these chemotherapeutic drugs are divided into alkylating agents which modify DNA directly, agents targeting cancer cell metabolism, topoisomerase inhibitors, and inhibitors of DNA repair pathways. Many of these drugs, often used in combination with other chemotherapies or radiation therapies, remain the mainstay of anti-cancer chemotherapies. Here, we review the DNA-damaging strategies currently used for cancer treatment in the clinic. It is important to note that, while DNA-damaging agents are generally effective in treating cancers, their clinical use is limited by their toxicity. In addition, drug resistance almost always arises. We review a potential strategy to address toxicity and drug resistance by targeting the protein proliferating cell nuclear antigen (PCNA), which plays a central role in the DNA replication and damage response network. In particular, the discovery of the cancer-associated isoform of PCNA (caPCNA) [7] and the development of small molecule and peptide agents that specifically target caPCNA allows for the selective targeting of cancer cells without causing significant toxicity to normal cells or experimental animals [8,9].

## 2. DNA Alkylating Agents

DNA alkylating agents, such as dacarbazine, cyclophosphamides, and Busulfan modify nucleotide bases, predominantly by attaching an alkyl group to DNA at guanine N7 [10]. Other sites of alkylation damage include N2 and O6 of guanine, N1 of adenine, and N3 of cytosine, which are also involved in the therapeutic responses of alkylating agents [11,12]. Some DNA alkylating agents contain dual reactive groups, which can alkylate two different bases and form interstrand and intrastrand crosslinks. For instance, Busulfan can form interstrand crosslinks between the DNA bases guanine and guanine and between guanine and adenine through its two labile methanesulfonate groups [13]. The prodrug cyclophosphamide can also form DNA crosslinks through its active metabolite, phosphoramide mustard [14]. Although DNA replication machinery can tolerate DNA lesions to a certain degree through various mechanisms [15,16], a high number of DNA lesions eventually collapse the DNA replication fork, leading to the formation of lethal DSBs. Because DNA alkylation agents react to DNA directly, their action is independent of the cell cycle. As a result, they are effective in treating a broad range of cancers [17].

Platinum analogs, including cisplatin, carboplatin, and oxaliplatin, exert their anti-cancer effects by a similar mechanism of action to that of alkylating agents [18]. Cisplatin, the first in this class that was approved as an anticancer agent, began to be used for the clinical treatment of testicular and bladder cancer in 1978 [19]. Inside cells, cisplatin is activated by hydrolysis in which its two chlorides are replaced with two water molecules [20]. This hydrolyzed product can bind to two DNA bases, predominantly at the N7 reactive site on purine residues on the same DNA strand [21]. These intrastrand crosslinks, or adducts, which block DNA replication, induce cell cycle arrest in the S and G2 phases to enable cells to repair the damaged DNA [22]. Nucleotide excision repair (NER) [23] and mismatch repair (MMR) [24] are involved in removing cisplatin adducts and repairing the DNA lesions. If the level of DNA damage overwhelms the repair systems, cells will die via apoptosis [25,26,27]. Cisplatin confers a significant survival benefit to cancer patients and is now broadly used in the treatment of leukemia, lymphoma, and many solid tumors such as breast, lung, gastric, and prostate cancers [28,29]. However, like most chemotherapeutic drugs, the use of cisplatin almost always leads to drug resistance, the mechanisms of which are mostly unclear [30,31]. In addition, cisplatin use is associated with systemic toxicities to bone marrow and to renal, gastrointestinal, and peripheral neural systems [32,33]. Up to one-third of patients receiving cisplatin treatment develop acute renal failure, and most patients suffering from cisplatin-induced renal dysfunction never fully recover [34].

## 3. Targeting Nucleotide Metabolism

Agents that target nucleotide metabolism, called nucleotide antimetabolites, are a group of purine or pyrimidine analogs that mimic the molecules cancer cells need to synthesize DNA, thus disrupting DNA synthesis. The effectiveness of antimetabolites in treating cancer is attributed to the increased metabolic demand of neoplastic cells, which leads to increased nucleotide biosynthesis and DNA replication. These drugs inhibit DNA replication mainly by depleting nucleotides, which, in turn, blocks DNA replication. Some nucleotide analogs can also be incorporated into newly synthesized DNA and block DNA replication fork progression. Notable examples include 5-fluorouracil (5-FU), a synthetic analog of uracil that inhibits thymidylate synthase [35]. Thymidylate synthase methylates deoxyuridine monophosphate into thymidine monophosphate. Administration of 5-FU limits the availability of thymidine nucleotides for DNA synthesis and induces cancer cell death. Administration of 5-FU by intravenous injection is widely used in the clinic to treat many solid tumors including breast, pancreatic, and gastrointestinal cancers [36]. It is also used topically for treating skin cancers [37,38]. In addition, a rationally designed oral prodrug [39] related to 5-FU, capecitabine, is also available for treating breast, pancreatic, and gastrointestinal cancers [40].

Another important antimetabolite drug is gemcitabine, which has been used as a chemotherapeutic drug for more than 20 years. Gemcitabine is a hydrophilic synthetic pyrimidine nucleoside prodrug, whose cellular uptake is catalyzed by a family of cell membrane nucleoside transporters including SLC28A1 (CNT1) and SLC29A1 (ENT1) [41,42]. Inside cells, gemcitabine undergoes several phosphorylation steps and is turned into the pharmacologically active gemcitabine triphosphate (dFdCTP) [43]. Gemcitabine has multiple modes of action inside cells. The most important action of gemcitabine is inhibiting DNA synthesis—it inhibits the enzyme ribonucleotide reductase (RNR), which is needed to create new DNA nucleotides, thereby inhibiting DNA synthesis [44,45]. dFdCTP can be incorporated into DNA, leading to the inhibition of DNA polymerases and preventing replication fork progression [46]. The inhibition of RNR reduces the dNTP pool in cells and further favors the incorporation of dFdCTP. These actions result in S and G2 cell cycle arrest. Gemcitabine chemoresistance and variations in its potency are common but not well understood. Mechanisms of chemoresistance likely involve multiple factors that affect gemcitabine transportation, activation, and metabolism [47,48].

## 4. Targeting Topoisomerase I/II

Topoisomerases (TOPOs) are nuclear enzymes required for normal DNA replication and cellular division. TOPO enzymatic activity rises significantly during DNA replication because of topological issues, such as overwinding of the DNA duplex, which must be released for DNA replication to continue. TOPOs are generally classified as type I or type II based on their mechanism of action [49,50]. Type I topoisomerase (TOPO I) is monomeric and makes single-strand DNA nicks that can untangle supercoiled double-stranded DNA and relax localized DNA torsional tension [42,51]. In contrast, Type II topoisomerase (TOPO II) is homo-dimeric or hetero-dimeric and addresses DNA topology issues by making double-stranded DNA breaks [52]. Once DNA cleavages are made, the TOPO enzymes are covalently linked to the 5′ or 3′ DNA phosphate [53,54]. Several TOPO inhibitors have been approved for treating colorectal, lung, ovarian, and hematological cancers. These drugs target this transitional cleavage intermediate. By stabilizing the TOPO-DNA covalent complex, they prevent the religation of DNA breaks and the progression of DNA replication, leading to cell death [55]. The compounds that function via such a mechanism are often referred to as TOPO poisons to distinguish them from those that inhibit the catalytic activity of TOPO. 

Both TOPO I and TOPO II are therapeutic targets for a broad spectrum of cancers [55]. TOPO I inhibitors currently in clinical use include topotecan, irinotecan, and belotecan. Topotecan is commonly used to treat metastatic ovarian cancer, cervical cancer, and small cell lung cancer, often in combination with other chemotherapeutic drugs, including cyclophosphamide, doxorubicin, and vincristine [41,56,57], while Belotecan is approved to treat small cell lung cancer. Irinotecan is a prodrug, and its anticancer effect depends on its conversion to the active metabolite, 7-ethyl-10-hydroxycamptothecin (SN38), by enzymatic cleavage of the C-10 side chain by carboxylesterase [58]. Irinotecan is approved to treat metastatic colon cancer. Much effort has been made to improve the delivery of irinotecan or SN38 and manage their side effects. This approach led to Onivyde^®^, a nanoliposomal form of irinotecan, which has been approved to treat pancreatic cancer [59]. By protecting irinotecan from premature metabolism in the plasma, this liposomal formulation enhances irinotecan activation and cytotoxicity in tumor tissue [60].

TOPO II inhibitors in clinical use include etoposide, teniposide, doxorubicin, and mitoxantrone. Derived from podophyllotoxin, etoposide, and teniposide act by trapping the TOPO/DNA covalent intermediate, leading to S and G2 cell cycle arrest and the accumulation of lethal DSBs [61,62]. Etoposide is a core agent of combination regimens for treating several cancers such as SLCL, lymphoma, and leukemia. Teniposide is currently used with other chemotherapy drugs for induction therapy to treat refractory acute lymphocytic leukemia in children. Doxorubicin and mitoxantrone, both anthracycline analogues, which can intercalate into DNA through their anthraquinone ring [63]. The resulting doxorubicin or mitoxantrone-DNA complex interferes with TOPO II enzyme activity and induces S and G2 cell cycle arrest and DNA damage. Instead of binding to TOPO II directly, doxorubicin and mitoxantrone inhibit TOPO II progression by DNA intercalation [63]. The planar aromatic rings of these types of compounds insert between two base pairs of the DNA and stabilize the TOPO II/DNA complex, preventing the DNA helix from unwinding during DNA replication and transcription. Mitoxantrone is administrated by intravenous injection and used to treat advanced prostate cancer and acute nonlymphocytic leukemia [64]. Doxorubicin, also administrated intravenously, is used to treat a broad range of cancers including breast cancer, bladder cancer, Kaposi’s sarcoma, lymphoma, and acute lymphocytic leukemia [65]. 

## 5. Targeting DNA Repair Signaling Pathways

**Figure 1:**
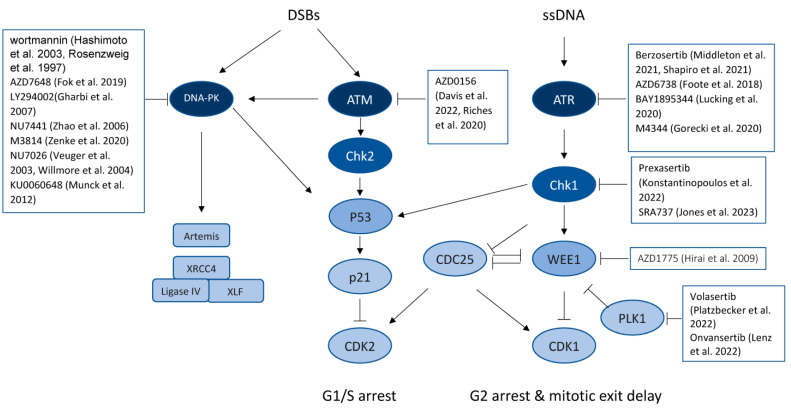
DNA damaging response signaling pathways [1,2,3,5,6,7,8,9,10,14,15,16,17,19,23,24,25,26,27,28,29]. PLK1: Polo Like Kinase 1; XRCC4: X-Ray Repair Cross Complementing 4; and XLF: XRCC4-like factor.

Mammalian cells have developed comprehensive mechanisms to sense and activate the DNA damage response (DDR), which is essential to maintain genome stability. The DDR is regulated by multiple cascades of kinase signaling pathways including the DNA-dependent protein kinase catalytic subunit (DNA-PKcs), ataxia telangiectasia mutated (ATM), and ATM and RAD3-related (ATR) pathways [66]. As shown in Figure 1, once activated by DNA damage, these kinase pathways activate checkpoint responses that arrest the cell cycle, allowing cells to repair or bypass damaged DNA sites and restart stalled or collapsed replication forks. Alternatively, if the DNA damage is beyond repair, then collapsed replication forks lead to lethal DSBs and cell death by apoptosis. Whereas the DNA-PKcs and ATM pathways mainly mediate the repair of DNA DSBs through the error-prone non-homologous DNA end joining (NHEJ) pathway [67] and the error-free homologous recombination (HR) pathway [68], the ATR pathway responds to DNA single-strand breaks (SSBs), and stalled DNA replication forks [69]. Targeting the DDR, therefore, enhances intracellular replication stress, stalled DNA replication, and lethal DSBs.

### 5.1. Targeting the DNA-PK Signaling Pathway

DNA-dependent kinase (DNA-PK) plays an essential role in the NHEJ pathway and interacts with multiple components of the DDR [66,70]. The catalytic subunit (DNA-PKcs) of DNA-PK, encoded by the *PRKDC* gene, belongs to the phosphatidylinositol 3 (PI 3)-kinase (PIKK) family, and is a DNA-activated serine/threonine protein kinase [71]. DNA-PKcs forms a holoenzyme DNA-PK with the heterodimer regulatory subunits Ku70 and Ku80. Ku70 and Ku80 [72,73], encoded by the *XRCC6* and *XRCC5* genes, respectively, detect DSBs by the Ku70/Ku80 heterodimer’s sequence-independent affinity [74] for available ends of double-stranded DNA. The binding of Ku70/Ku80 to the ends of DSBs maintains the stability of broken ends and initiates NHEJ. Ku70/80 is responsible for recruiting canonical NHEJ factors such as DNA-PKcs, XRCC4, XFL, and DNA ligase IV to the broken ends of DNA [75]. The interaction of DNA-PKcs with the Ku70/Ku80 heterodimer leads to a direct interaction of DNA-PKcs with DSB ends and activation of the kinase activity of the DNA-PKcs [72,76]. DNA-PKcs is regulated by auto-phosphorylation [71] as well as phosphorylation by ATM [77]. Auto-phosphorylation can cause a conformational change in DNA-PKcs, which allows for DNA end processing [78,79]. After sensing DNA damage, cells face the choice of using the more efficient but error-prone NHEJ or the less efficient but error-free HR pathway to repair DNA damage [80]. DNA-PK can inhibit HR activity, thereby favoring NHEJ [81]. 

Given its role in multiple DDR nodes, DNA-PKcs has become an attractive anti-cancer therapeutic target, especially in combination with genotoxic chemotherapy or radiation therapy. Many small molecule inhibitors of DNA-PKcs are currently under development through clinical trials (Figure 1). These compounds range from the early pan PIKK family kinase inhibitor wortmannin [82,83] to selective DNA-PKcs inhibitors such as AZD7648 [84] and M3814 [85]. AZD7648, a potent and highly selective DNA-PKcs inhibitor, works efficiently to sensitize cancer cells to ionizing radiation and doxorubicin and induces sustained tumor regressions in animal models. AZD7648 also works synergistically with the PARP inhibitor olaparib to inhibit cell growth inhibition and induce apoptosis [84]. M3814, another DNA-PKsc selective inhibitor, also sensitizes cells to chemotherapeutic agents, including anti-microtubule drugs such as paclitaxel and topoisomerase II inhibitors such as daunorubicin [86]. In mouse tumor models, M3814 augments the antitumor effects of chemotherapeutic agents such as calicheamicin, paclitaxel, etoposide, pegylated liposomal daunorubicin, and 5-fluorouracil [87,88,89]. Of the DNA-PK inhibitors in Figure 1, M3814 and AZD7648 (Table 1) are in clinical trials as monotherapies and in combination with radiation or other chemotherapies [90]. 

### 5.2. Targeting ATM/CHK2 and ATR/CHK1 Signaling

After sensing DNA damage, it is paramount to arrest cell cycle progression to allow cells time to repair the damaged sites and thereby maintain genomic stability. The ATM/CHK2 and ATR/CHK1 kinase cascades are the two main signaling pathways regulating cell cycle arrest during DDR [66,99,100] (Figure 1). Like DNA-PKcs, both ATM and ATR are members of the PIKK family. When a DSB occurs, ATM is activated in the presence of the Mre11–Rad50–NBS1 (MNR) complex through auto-phosphorylation [101,102]. ATM relays and amplifies the signal from MNR by phosphorylating its substrate enzymes, including Checkpoint Kinase 2 (CHK2), which, in turn, phosphorylates transcription factor p53 [103]. The ensuing p53-dependent upregulation of cyclin-dependent kinase inhibitor 1 (p21Cip1) leads to the activation of the retinoblastoma (RB) protein and G1 arrest [104]. ATR is involved in a broad spectrum of DDR and is activated by DSBs and ssDNA [66,69] as well as DNA crosslinks [105]. During ssDNA repair, ATR and ATR interacting protein (ATR-ATRIP) complex is recruited to the ssDNA site and is activated by the hetero-trimetric Rad9-Rad1-Hus1 clamp that is loaded onto 5′-recessed DNA by Rad17-RFC [106]. Many ATR functions are mediated through its downstream target Checkpoint Kinase 1 (CHK1), which mediates the phosphorylation of the cell division cycle 25 (Cdc25) family phosphatases and Wee1-like protein kinase (WEE1), leading to G2 cell cycle arrest, which is pivotal for premitotic DNA repair [107,108]. In this context, numerous efforts were made to develop inhibitors of the ATM/CDK2 and ATR/CHK1 pathways for targeted therapy against cancer (Figure 1 and Table 1).

### 5.3. Inhibiting WEE1

WEE1 is a serine/threonine kinase that plays a key role in regulating cell cycle progression (Figure 1). Wee1 activation by Chk1 inhibits cyclin-dependent kinase 1 (CDK1), a key G2/M checkpoint regulator that is required for cyclin-dependent entry into mitosis [109,110]. Genotoxic stress is common in cancer cells because of endogenous factors such as reactive oxidative species, compromised DNA repair capacities, and the loss of G1 checkpoint control due to oncogene actions or the loss of tumor suppressor genes. As a result, cancer cells rely on WEE1 activity to initiate G2/M arrest and to provide time for DNA damage repair. Inhibition of WEE1 prevents G2/M cell cycle arrest, leading to premature mitotic entry with unrepaired DNA damage and subsequent cell death [109,110,111]. WEE1 also protects replication forks and inhibition of WEE1 can induce the uncontrolled firing of replication origins, leading to increased replication stress [112,113]. Given these effects, several WEE1 inhibitors (Figure 1 and Table 1) have been developed with a focus on engineering synthetic lethality by using WEE1 inhibitors in combination with DNA-damaging chemotherapies or radiation [114,115]. Importantly, greater than 50% of all human cancers harbor mutations in the tumor suppressor gene p53, which plays a major role in genomic stability by transcriptionally regulating downstream genes involved in the G1/S checkpoint in response to DNA damage [116]. Preclinical studies found that abrogation of the G2 checkpoint by WEE1 inhibition can sensitize p53-deficient cells to chemotherapies and radiation, leading to mitotic catastrophe [109,110,111]. The most advanced WEE1 inhibitor in development, AZD1775 (adavosertib) [117], is currently being investigated in more than a dozen clinical trials targeting lung (NCT02513563), ovarian (NCT01164995, NCT02101775, and NCT03579316), renal (NCT03284385), pancreatic (NCT02101775), uterine (NCT03668340), bladder (NCT02546661), cervical (NCT03345784), hematopoietic (NCT04439227), and neural (NCT02095132 and NCT01849146) cancers. Many of these studies evaluate the effect of AZD1775 on p53-deficient cancers (NCT01164995, NCT02101775, NCT03668340, and NCT01849146), BRCA-deficient cancers (NCT04439227), and/or in combination with DNA-damaging agents (NCT02513563, NCT01164995, NCT02101775, and NCT03579316, NCT02101775, NCT03668340, NCT02546661, NCT03345784, NCT02095132, and NCT01849146). AZD1775 analogs with reduced cellular cytotoxicity have been reported to address its dose-limiting toxicities including neutropenia, thrombocytopenia, anemia, diarrhea, fatigue, and vomiting [118]. An AZD1775-based WEE1 degrader (ZNL-02-096) reportedly shows distinct pharmacology than AZD1775 in preclinical development [119].

## 6. Targeting DNA Repairing Proteins

### 6.1. Targeting the PARP Pathway

**Figure 2:**
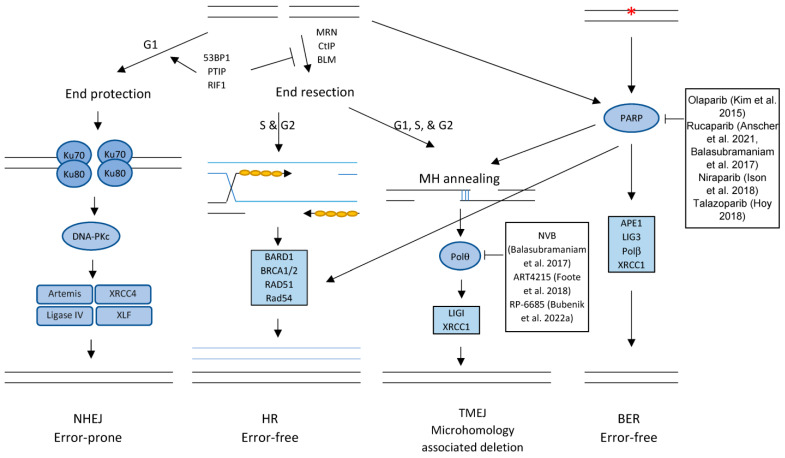
DNA repair pathways for DSBs and SSBs [4,11,12,13,15,18,20]. 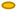 RPA and 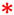 DNA damage. MRN: Mre11/Rad50/Nbs1 complex; 53BP1: p53-binding protein 1; PTIP: Pax transactivation domain-interacting protein; RIF1: Replication Timing Regulatory Factor 1; CtIP: CtBP (carboxy-terminal binding protein) interacting protein; LIGI: DNA ligase 1; LIG3: DNA ligase 3; XRCC4: X-ray Repair Cross Complementing 4; XLF: XRCC4-like factor; APE1: DNA (apurinic/apyrimidinic site) endonuclease 1; Polβ: DNA polymerase β; BARD1: BRCA1 associated RING domain 1; MH: microhomology.

Poly (ADP-ribose) polymerase (PARP) is a family of multi-function proteins that play roles in DNA repair and genome integrity [120]. The family consists of 17 members [121], among which PARP-1 is the most abundant in cells and plays dominant roles in regulating DNA repair [122]. PARP-1 is critical for SSB repair and base excision repair (BER). PARP-1 binds to SSB and activates its enzymatic activity to synthesize a poly (ADP-ribose), or PAR, on itself and other DNA repair proteins including DNA ligase 3, DNA polymerase β (polβ), and XRCC1 [123]. Therefore, PARP-1 is critical to the recruitment of DNA repair proteins to the damaged sites (Figure 2). In addition to its role in SSB and BER repairs, PARP-1 supports DSBs repair in multiple ways, including the recruitment of MRE11 and NBS1 to the damage sites [124], the transcriptional regulation of BRCA1 and Rad51 [125], both of which play important roles in the HR pathway, and regulation of BRCA1 function [126]. Several PARP inhibitors gained FDA approval for treating cancers that harbor mutated BRCA1 or BRCA2 genes [93,97,98,127,128]. BRAC1 and BRCA2 are both involved in repairing DSBs by the HR pathway [129]. It is widely accepted that PARP inhibition blocks SSB repair and causes DSBs to form. In cells deficient in BRCA1 or BRCA2, these DSBs cannot be efficiently repaired, due to an impaired HR pathway [130]. Therefore, cancer cells harboring mutated BRCA1 or BRCA2 are particularly sensitive to PARP inhibition [130,131]. Numerous studies have also demonstrated that PARP inhibition enhances the anti-cancer therapeutic effect of other chemotherapeutic drugs and radiation [132,133,134,135]. Combination therapies involving PARP inhibitors in combination with bevacizumab, paclitaxel, cisplatin, topotecan, carboplatin, or gemcitabine are currently in clinical trials [136].

### 6.2. Inhibiting DNA Polymerase Theta

DNA polymerase theta (POLθ) plays a key role in theta-mediated end joining (TMEJ) [137,138], which is one of three DSB repair mechanisms (Figure 2). DSBs are predominantly repaired by the NHEJ repair pathway during the G1 phase and by the HR pathway during the S/G2 phases of the cell cycle. TMEJ is considered the only “backup” DSB repair solution and is used when the NHEJ or HR response is insufficient or compromised [139]. Importantly, POLθ is not significantly expressed in normal cells, but its expression is increased in many cancers [140,141]. Patients whose tumors overexpress POLθ are often associated with poor prognosis [142,143], possibly because the error prone TMEJ activity could result in increased genetic diversity among tumor cells and increase the chance of the development of drug resistance. Furthermore, cancer cells deficient in the HR or NHEJ pathway, or deficient of the ATM kinase depend heavily on MMEJ and are especially sensitive to POLθ disruption [142,143,144]. This phenomenon provides a strong rationale for engineering synthetic lethality by inhibiting POLθ in cancer cells harboring other DSB repair defects. Several Polθ inhibitors (Table 1) have been reported [91,145,146], and two of them, ART4215 [146] and the antibiotic NVB [145], are being tested in clinical trials against HR-deficient tumors. NVB is a coumarin antibiotic and was discovered in a small molecule screen for inhibitors of POLθ ATPase activity. NVB binds to purified POLθ protein, prevents its recruitment to DNA damage, and inhibits TMEJ repair. Importantly, NVB selectively kills cancer cells harboring HR deficiency (BRCA1- and BRCA2-deficiency) and potentiates the cytotoxic effect of PARP inhibition in HR-deficient cancer cells [145]. Moreover, NVB kills HR-deficient tumor cells, which have acquired resistance to PARP inhibitors [145], demonstrating its therapeutic potential in combination with PARP inhibition for treating HR-deficient tumors.

### 6.3. Inhibiting RECQ Helicases

The evolutionarily conserved RecQ helicase family enzymes drive the unwinding of DNA strands in the 3′ to 5′ direction and play important roles in genome maintenance including DNA replication, DNA repair, transcription-associated stress management, and telomere maintenance [147]. Humans have five RecQ helicases: RECQL1, Bloom syndrome protein (BLM), Werner syndrome helicase (WRN), RECQL4, and RECQL5 [148]. Defects in RecQ helicase are associated with several genetic disorders including a predisposition to tumorigenesis [148,149]. For instance, dysfunctional mutations in RECQL5 are associated with a susceptibility to breast cancer [150], head and neck cancer [151], and gastric cancer [152]. Importantly, RecQ helicases are essential to the repair of DNA DSBs. For instance, RECQL4 helicase promotes HR repair in the S and G2 cell cycle phases and facilitates NHEJ through functional interaction with Ku70/Ku80 in the G1 phase [153,154,155]. Collectively, they are essential to each of the DSB repair pathways including the NHEJ, HR, and alternative NHEJ pathway, which ligates DSB ends without the use of extensive homology and in a Ku70/Ku80 independent manner [156]. Therefore, this family of enzymes provides a unique panel of potentially predictive biomarkers to choose therapeutic agents that target DNA repair pathways. Preclinical studies also suggest that RecQ helicase inhibitors are likely to work synergistically with other DNA-damaging agents to kill cancer cells [157,158,159,160].

## 7. Targeting PCNA, the “hub” Protein of DNA Replication and Repair Networks

The ultimate challenge for cancer treatment is to selectively kill cancer cells while sparing normal tissue. Many traditional chemotherapeutic drugs exploit the intrinsic addiction to DNA repair machinery [5,6] and induce cell death by overloading replication stress through their DNA damaging properties. Although effective initially, these DNA-damaging agents cause severe side effects and often induce drug resistance [161], both of which limit their long-term use in the clinic. In addition, the mechanism or mechanisms leading to drug resistance are mostly unclear. More recently, many therapeutic agents that target specific oncogenic signaling components, including cell cycle checkpoints, have reached the clinic [162,163,164,165,166,167]. Although causing less severe side effects than early chemotherapeutic agents such as cisplatin in general, the success of these target-based therapies is limited by the rapid development of drug resistance [168,169,170] through the accumulation of mutations within target genes or by the activation of alternate survival pathways. One promising strategy to prevent such acquired drug resistance, which is inherent in the adaptive and heterogeneous nature of cancers, is to target “hub” proteins whose functions are central to broad and essential cellular processes. The key is to target crucial processes, such as the DNA replication or repair process, without causing unacceptable side effects in non-malignant cells. Recent studies of PCNA provide proof of concept of this promising strategy. PCNA is an evolutionally conserved protein found in all eukaryotic cells. Forming a homotrimeric ring structure encircling DNA [171,172], PCNA acts as a central “hub” to provide anchorage for more than a dozen proteins [173], mainly through its interdomain connector loop (IDCL) that spans from amino acid M121 to Y133 [171]. Proteins that interact with this loop include p21 (CDKN1A) [174], DNA polymerase δ (Pol δ) [175], flap endonuclease 1 (FEN1) [176], DNA methyltransferase (MeCTr) [177], and DNA ligase 1 (LIGI) [178], which interact with PCNA through their PIP-box domains [173,179]. In addition to recruiting these proteins to chromatin, PCNA provides a sliding “working platform” for these proteins to regulate DNA replication, cell cycle progression, and DNA damage responses [180]. Because of PCNA’s fundamental role in cell growth, survival, and mutagenesis, many attempts have been made in recent years to therapeutically inhibit PCNA with promising results [174,181,182,183], demonstrating the potential of PCNA as a therapeutic target for cancer treatment.

Importantly, a novel cancer-associated PCNA isoform (caPCNA) was discovered to be the predominant PCNA isoform expressed in a broad range of cancer cells and tumor tissues but was not highly expressed in non-malignant cells [7]. The caPCNA isoform was not caused by a genetic mutation or alternative mRNA splicing but arose as a result of posttranslational modification [184] that affects the protein structure and the accessibility of the L126-Y133 region within the IDCL of PCNA [7]. A cell permeable peptide (R9-caPep) containing the L126-Y133 sequence of PCNA selectively blocks PCNA interactions in cancer cells and interferes with DNA synthesis and HR-mediated DSB repair, resulting in S-phase arrest, an accumulation of DNA damage, and an enhanced sensitivity to cisplatin [9]. R9-caPep also selectively kills cancer cells with much less toxicity to human peripheral blood mononuclear cells or neural crest stem cells and suppresses cell growth in a mouse xenograft model without causing any discernable toxicity to the animals [9,185,186,187]. These findings demonstrate that targeting protein–protein interactions involving the L126-Y133 region of PCNA may prove to be an effective approach to treating cancers with reduced side effects.

Small molecule compounds, AOH1160 and its analogs, have also been developed to target the caPCNA protein–protein interaction region [8]. AOH1160, which binds to a PCNA surface pocket partly delineated by the L126-Y133 region, interferes with DNA replication and blocks HR-mediated DNA repair, leading to cell cycle arrest, the accumulation of unrepaired DNA damage, and an enhanced sensitivity to cisplatin treatment [8]. A biologically stable analog of AOH1160, AOH1996, was developed to be orally available to animals and suppresses tumor growth without causing significant side effects in mice (unpublished results). AOH1996 is currently in a clinical trial (Phase 1 Study of AOH1996 in Patients with Refractory Solid Tumors Protocol Type: Treatment, NCT ID: NCT05227326).

## 8. Challenges and Future Perspectives

A major challenge of anticancer chemotherapy is chemoresistance [188]. Some tumors are refractory to drug treatment. The development of acquired resistance is common for all existing chemotherapeutic regimes, which leads to disease reoccurrence. In addition, the complexity of cancers also presents a significant clinical hurdle: how can we effectively treat such diseases arising from varied and continual mutagenesis? Targeting proteins that act as central “hubs” of cellular processes that are essential to dealing with cancer-specific stresses may provide a novel strategy to overcome drug resistance. In addition to the need to maintain genome stability, the survival of cancer cells depends on additional pathways to deal with proteotoxic stress, mitotic stress, metabolic stress, and oxidative stress. Although these pathways play normal and often ubiquitous cellular functions, many rate-limiting proteins in these pathways are essential for dealing with the increased stresses of cancer cells [6]. Unlike oncogenes, these non-oncogenic target genes do not undergo oncogenic mutations or functionally significant genomic alterations in tumors and thus represent points of intervention that are less prone to the development of resistance. Cancer drug discoveries targeting these non-oncogenic pathways have yielded a number of successful therapeutics [189,190,191]. As exemplified by the discovery of caPCNA, which led to the development of first-in-class small molecules with superior anti-cancer properties, AOH1160 and AOH1996, future studies to identify cancer-specific features of critical, functional nodes in these networks may lead to safer and more effective therapies to treat cancer.

## Figures and Tables

**Table 1:** Major anti-DNA repair therapeutics approved for cancer treatment or in advanced development.

Target	Agent	Cancer Type	Phase
ATR	Berzosertib	Lung Cancer	Phase II (Sources: clinicaltrials.gov) Access date: 30 May 2023
	AZD6738	Bile duct cancer Clear cell renal cell carcinoma Breast cancer	Phase II (Sources: clinicaltrials.gov) Access date: 30 May 2023
	BAY1895344	Advanced solid tumor Non-Hodgkin’s lymphoma Mantle cell lymphoma	Phase I (Sources: clinicaltrials.gov) Access date: 30 May 2023
	M4344	Recurrent ovarian cancer	Phase I (Sources: clinicaltrials.gov) Access date: 30 May 2023
Chk1	Prexasertib	Ovarian cancer Triple-negative breast cancer Small cell lung cancer	Phase II (Sources: clinicaltrials.gov) Access date: 30 May 2023
	SRA737	Advanced solid tumors Non-Hodgkin’s lymphoma	Phase 1/II (Sources: clinicaltrials.gov) Access date: 30 May 2023
WEE1	AZD1775	Advanced solid tumor Refractory solid tumor Triple-negative breast cancer Ovarian cancer Pancreatic cancer	Phase II (Sources: clinicaltrials.gov) Access date: 30 May 2023
PLK1	Volasertib	Myeloid acute leukemia	Phase III (Sources: clinicaltrials.gov) Access date: 30 May 2023
	Onvansertib	Colorectal cancer Breast cancer Pancreatic cancer Small cell lung cancer	Phase II (Sources: clinicaltrials.gov) Access date: 30 May 2023
DNA-PK	AZD7648	Advanced malignancies	Phase I (completed) (Sources: clinicaltrials.gov) Access date: 30 May 2023
	M3814	Pancreatic cancer Prostate cancer Locally Advanced Rectal Cancer	Phase II (Sources: clinicaltrials.gov) Access date: 30 May 2023
DNA polymerase theta	NVB	Tumors That Have Alterations in DNA Repair Genes	Phase I (Sources: clinicaltrials.gov) Access date: 30 May 2023
	ART4215	Advanced or Metastatic Solid Tumors	Phase I/II (Sources: clinicaltrials.gov) Access date: 30 May 2023
	RP-6685	BRCA-mutant breast and ovarian cancers	Preclinical development [91]
PARP	Olaparib	BRCA-mutant breast cancer Ovarian cancer Prostate cancer	Approved drug [92,93]
	Rucaparib	BRCA-mutant prostate cancer Recurrent Ovarian Cancer BRCA-mutant Ovarian cancer	Approved drug [94,95,96]
	Niraparib	Epithelial ovarian, Fallopian tube, or primary peritoneal cancer	Approved drug [97]
	Talazoparib	BRCA-mutant HER2-negative breast cancer	Approved drug [98]

## Data Availability

Not applicable.
